# An Anomalous Noise Events Detector for Dynamic Road Traffic Noise Mapping in Real-Life Urban and Suburban Environments

**DOI:** 10.3390/s17102323

**Published:** 2017-10-12

**Authors:** Joan Claudi Socoró, Francesc Alías, Rosa Ma Alsina-Pagès

**Affiliations:** GTM—Grup de recerca en Tecnologies Mèdia, La Salle, Universitat Ramon Llull, Quatre Camins, 30, 08022 Barcelona, Spain; falias@salleurl.edu (F.A.); ralsina@salleurl.edu (R.M.A.-P.)

**Keywords:** real-time acoustic event detection, anomalous noise events, road traffic noise, background noise, dynamic noise mapping, wireless acoustic sensor network, binary classification, real-life audio database, urban and suburban environments

## Abstract

One of the main aspects affecting the quality of life of people living in urban and suburban areas is their continued exposure to high Road Traffic Noise (RTN) levels. Until now, noise measurements in cities have been performed by professionals, recording data in certain locations to build a noise map afterwards. However, the deployment of Wireless Acoustic Sensor Networks (WASN) has enabled automatic noise mapping in smart cities. In order to obtain a reliable picture of the RTN levels affecting citizens, Anomalous Noise Events (ANE) unrelated to road traffic should be removed from the noise map computation. To this aim, this paper introduces an Anomalous Noise Event Detector (ANED) designed to differentiate between RTN and ANE in real time within a predefined interval running on the distributed low-cost acoustic sensors of a WASN. The proposed ANED follows a two-class audio event detection and classification approach, instead of multi-class or one-class classification schemes, taking advantage of the collection of representative acoustic data in real-life environments. The experiments conducted within the DYNAMAP project, implemented on ARM-based acoustic sensors, show the feasibility of the proposal both in terms of computational cost and classification performance using standard Mel cepstral coefficients and Gaussian Mixture Models (GMM). The two-class GMM core classifier relatively improves the baseline universal GMM one-class classifier F1 measure by 18.7% and 31.8% for suburban and urban environments, respectively, within the 1-s integration interval. Nevertheless, according to the results, the classification performance of the current ANED implementation still has room for improvement.

## 1. Introduction

Continued exposure to high traffic noise levels has been proven to cause harmful effects on health, considered to be one of the main aspects that affect the quality of life of people living in urban and suburban areas [1]. To address this issue, local, national and international authorities have conducted several initiatives to measure, prevent and reduce the effects of human exposure to traffic noise. Among them, the European Noise Directive 2002/49/EC (END) [2] and the consequent strategic noise mapping assessment Common Noise Assessment Methods in Europe (CNOSSOS-EU) [3] are the main instruments at the European level concerned with noise pollution and the elaboration of the suitable action plans, as well as being responsible for informing and consulting the concerned population appropriately. The END requires European member states to prepare and publish both noise maps and the corresponding noise management action plans [2] every five years.

Noise measurements in cities have been conventionally performed by professionals who record and analyze the data in specific locations, using certified sound level meters. From the gathered data, noise maps are generated by means of complex acoustic models after data post-processing, which typically consists of a traffic noise source intensity emission model plus a sound propagation model [4]. However, this approach becomes difficult to scale when more frequent measurements are needed or when more locations are required. Recent technological advances within the smart city and the Internet of Things frameworks have allowed a change of paradigm for city noise management through the deployment of Wireless Acoustic Sensor Networks (WASN) [5]. In the last decade, several works focused on the design and implementation of WASN for environmental noise mapping have been proposed [6]. The main goal of these approaches has been to develop affordable solutions, maintaining the reliability of the acoustic measures, while improving the scalability of the system by means of optimizing the network design. Some WASN-based systems have been developed and tested across Europe, such as the IDEA project (Intelligent Distributed Environmental Assessment) in Belgium [7], the RUMEURwireless network [8] and the Cense project [9] in France, or the `Barcelona noise monitoring network’ in Spain [10], which follows quite similar approaches. Moreover, the SONYC project (Sounds of New York City) [11], the goal of which is to monitor the noise pollution in New York City, also provides an accurate description of its acoustic environment. A more recent initiative is the DYNAMAP project (DYNamic Acoustic MAPping), which aims to develop a dynamic noise mapping system able to detect and represent the acoustic impact of road traffic noise in Italy in real time [12,13,14], taking advantage of previous conclusions obtained by the SENSEableproject, focused on the collection of information about the acoustic environment using low-cost acoustic sensors [15].

The deployment of these WASN in several European countries has opened up some challenges [16,17], especially those derived from network and hardware platform design or data collection plus their subsequent audio signal processing (see [5,18] for further details). For instance, it is worth mentioning that none of the approaches described in [6] take into account the identification of traffic typology (e.g., light or heavy), nor the detection of specific acoustic events of interest (e.g., an air-craft flying over the road, nearby railways, road works, bells, crickets, etc.), which are necessary to cope with the END demands of noise source distinction [2].

In order to identify these kinds of events, it is necessary to determine the acoustic event start and end points within continuous audio streams [19]. To tackle this goal, Acoustic Event Detection (AED) follows two main approaches, in general terms, namely detection-and-classification and detection-by-classification [20]. Although the former allows for the detection of sudden changes within the audio stream with respect to the background noise (typically based on events’ acoustic salience), it does not identify the sound source. On the contrary, the latter enables recognition of the input acoustic data in a predefined closed set of acoustic classes or audio events, after enough representative data have been collected to train the classifier appropriately. The multi-class detection-by-classification AED has provided good results in different domains of application, such as ambient assisted living [21,22], birdsong recognition [23,24] or outdoor monitoring [25,26,27], to name a few. Moreover, the research on AED in urban environments has attracted much attention in recent years, mainly focused on surveillance applications, which may also include noise source identification [28]. Recent research about this topic has been centered on the introduction of specific metrics for polyphonic AED [29], on the implications of building real-life acoustic databases [30], as well as the application of deep learning to multi-class AED-related problems, provided enough training data are available (see [31] and references therein). Nevertheless, since these works are mainly focused on addressing signal processing challenges, most of them ignore the analysis of the applicability of the proposals on WASN or opt for implementing and running the algorithms on centralized servers [26,32], yielding burdensome communication costs that could be drastically reduced if those algorithms ran in the WASN low-cost acoustic sensors.

In order to provide public bodies with a reliable picture of the Road Traffic Noise (RTN) levels affecting citizens [2], as a first step to addressing the END demands, events unrelated to RTN, denoted as Anomalous Noise Events (ANE), should be removed from the noise map computation [33,34]. However, the identification of ANE from RTN and/or Background noise (BCK) is very challenging [12,34,35] due to the highly local, occasional, diverse and unpredictable nature of ANE in real-life scenarios [33,35,36], which makes the ANE class unbounded [37]. As a consequence, it becomes unfeasible to tackle the problem at hand through a multi-class detection-by-classification AED approach [36], as it operates on a predefined closed set of a priori known acoustic classes [38,39] after being trained with enough data of each class. As an alternative when sufficient samples are not available, some investigations have opted to build representative databases synthetically for a specific set of ANE by considering different Signal-to-Noise Ratios (SNR) between ANE and RTN/BCK to enable the multi-class AED to perform properly, e.g., see [34,35]. Although this approach can help to improve the results of the AED in a closed multi-class train-and-test evaluation scheme [33,35], it yields a negative impact on the system performance when tested in real-life conditions due to acoustic data misrepresentation [40]; e.g., the synthetic database considering only ANE with +6 dB and +12 dB as in [33] or including SNRs ranging from −5 dB–15 dB with a 5-dB step as in [27] do not represent what is actually found in real-life environments with a larger diversity and with significantly lower average SNRs [30]. Otherwise, the novelty detection approach may overcome multi-class AED by exclusively focusing on the major acoustic class (i.e., the one composed of the non-anomalous or predominant sound events) by means of One-Class Classification (OCC). The reader can see [41] and the references therein for a comprehensive review about novelty detection. This approach has been applied to detect aircraft-related noise events within an airport [42], as well as to implement surveillance applications in urban environments (e.g., detecting gun-shots, broken glasses, screams, etc.) [36,43], and the results have been good. However, this alternative lacks the information of ANE that could be of use for the detection system if enough data are available.

The purpose of this paper is to take advantage of the collection of representative real-life acoustic data to develop an Anomalous Noise Events Detector (ANED) capable of running on the low-cost sensors of a WASN distributedly (i.e., not running in a centralized remote server and working with raw acoustic data) in real time. To that effect, the ANED is designed as a two-class classifier (ANE vs. RTN) whose outputs are obtained after a two-level decision process: the frame-level binary decisions are integrated through a high-level decision based on a majority vote computed in a predefined interval. This proposal has been developed and validated on the suburban and urban pilot areas of the DYNAMAP project, fulfilling the computational requirements of its ARM-based acoustic sensors [44].

This paper is organized as follows. First, Section 2 reviews previous works focused on acoustic urban sensing and the application of acoustic event detection in urban environments. Secondly, the description of the proposed ANED is detailed in Section 3. Section 4 describes the conducted experiments considering real-life data from the two analyzed environments (suburban and urban). Section 5 discusses the most relevant aspects derived from this work regarding open research issues. Finally, Section 6 presents the main conclusions and future work directions.

## 2. Related Work

In this section, we describe representative approaches developed to automatically measure the noise levels of the cities in order to tailor noise maps [6]. The first part reviews the platform design or hardware approach of several relevant noise mapping projects. The second part reviews the acoustic event detection approaches introduced in the literature to face the automatic detection of acoustic events in urban environments, paying special attention to the works that try to consider real-life operating scenarios.

### 2.1. Static Acoustic Urban Sensing Platforms and Their Applications

In this section, several static noise sensing platforms and their applications are summarized, with particular attention paid to those whose main focus is the monitoring of environmental noise on specific locations in urban areas.

The first category of acoustic sensor networks is built for high accuracy and reliability and a low noise floor; so, usually, the price of the deployment of a network with several nodes is not an affordable option for its purpose. Most of these acoustic sensors are monitoring stations from Bruel and Kjaer [45] or Larson and Davies [46] and are equipped with IECClass 1 microphones. In the literature, some projects can be found that work with sensors of this category, and their goal is to perform a detailed study of the acoustic signal recorded in the city. In [26,32], the FI-Sonic project based on the FIWAREplatform is described. The project consists of an acoustic sensor network based on ambisonics microphones, a multichannel acquisition card (from 2–128 GB), a network interface (with a Wi-Fi/3G modem) and a centralized server as its main processing unit that runs the audio analyses. The information collected is used to create quasi-real-time dynamic noise and event maps, as well as to identify specific pre-trained sound sources. The main application of the project is focused on surveillance, ranging from the localization of gun shots to the identification of people in distress.

A second category of acoustic sensor networks is designed to balance the accuracy and the price of each node in the network. They are usually designed to be deployed in large networks, and their goals are not limited to price and accuracy, but also to take into account the need of additional acoustic processing of the dedicated hardware in each node of the network. Some of the networks in this category are based on commercial sound level meters, such as the systems designed by Wang [47], a project designed to monitor the traffic noise in Xiamen City (China). The designed system considers noise meters, ZigBee technology and GPRS communication. In [48], the authors also prefer to use a customized noise level meter, which is designed to remove the burden of computational and energy expensive operations from the sensor node and process them in the cloud, which is an usual design decision when the computational load of the required signal processing is unaffordable. Furthermore in [49], the authors obtain the measurements of the RUMEUR project from sound level meters, in a sensor network that pursues understanding measured phenomena, assesses actions against noise and communicates the information about the sound environment in Ile-de-France.

Nevertheless, some of the acoustic sensor designs of this second category are developed ad hoc for each application, taking into account the trade-off between the price and the accuracy. In [50], the urban sound environment of New York City is monitored using a low-cost static acoustic sensing network; it is a scalable system due to its low price per node, and the microphones are Class 2 MEMdevices. The processing core of the sensor is a mini PC Cortex A9 processor, which efficiently implements the digital signal processing required routines. In [17], the authors present the design of an acoustic sensor network using three main components: basic nodes, advanced nodes and a server. The hardware platform for a basic node is a low power microcontroller (μC) whose main goal is to transmit the collected data. The advanced nodes are capable of far more processing in comparison with the basic ones; they use a small PC with a 2-GHz Intel Atom Processor running a Linux operating system. The advanced node can both store and process data, and it is designed to be flexible for the signal processing analyses.

The cost versus accuracy balance is also the core idea of the DYNAMAP project sensor network design [12,44], aimed at the deployment of a low-cost WASN in two pilot areas: one in Rome [14] and the other in Milan [13]. The sensors designed for the DYNAMAP project are low-cost and use Class 2 MEM microphones. The WASN will deploy two types of sensors: (i) high capacity ARM-based sensors, allowing signal processing techniques to analyze and process the acoustic signals in each node [44]; and (ii) low capacity microcontroller-based sensors, with less computation capabilities and fed by solar panels, but more flexible in terms of sensor positioning, maximizing the coverage of the network. The DYNAMAP can be considered a step forward after the preliminary results obtained by the SENSEable project deployed in the city of Pisa [15]. SENSEable proposed a WASN to collect information about the acoustic environment of the city using low-cost acoustic sensors to study the relationship between public health, mobility and pollution through the analysis of citizens’ behavior. Finally, in this sensor category, one can already find some commercial solutions at a slightly higher price, but with less flexibility, such as Libelium Waspmote Plug&Sense [51], which measures simple dBA values with no type certification; the system can transfer data wirelessly to a central server, and it is well prepared for outdoors operation.

Finally, we can find a third category of acoustic sensor networks also designed with a balance between cost and accuracy, but with the possibility of being deployed in a pervasive manner as they are not static WASNs. Two of the projects using this kind of network are the IDEA project [7] and the MESSAGEproject [52]. They are based on a single board computer with low computational capacity, working with a low-cost sound card and microphones. This hardware choice permits the deployment of large sensor networks due to the low economic cost of each node, besides allowing the collection of relevant environmental data from several critical locations in the city. In the IDEA project [53], a cloud-based platform is also developed by integrating an environmental sensor network with an informative web platform and aims to measure noise and air quality pollution levels in urban areas in Belgium.

### 2.2. Detecting Acoustic Events in Urban Environments

In this section, we review the most significant works focused on acoustic event detection in urban environments, differentiating one-class novelty detection from multi-class-based classification approaches. The former are only focused on detecting the events of interest as different from the majority class. The latter are trained to detect a closed set of previously defined acoustic classes of interest. The section also includes several papers addressing the identification of specific hazardous situations related to traffic noise for surveillance applications. The reader is referred to [37] for a complete review of audio-based surveillance.

#### 2.2.1. One-Class Novelty Audio Event Detection

Ntalampiras et al. describe a probabilistic novelty detection approach for acoustic surveillance under pseudo-real-life conditions [36]. The study includes normal and abnormal (or anomalous) audio events such as screams, shouting or pleading for help, which are collected in two outdoor public security scenarios. This work is an evolution of a previous study by the same authors based on an artificially mixed environmental database (with SNRs ranging from −5 dB–15 dB with a 5-dB step) in a metro station environment [27]. The acoustic data are parameterized every 30 ms using a multi-domain feature vector including different audio descriptors, such as Mel Frequency Cepstral Coefficients (MFCC), MPEG-7 Low-Level Descriptors (LLD), intonation and Teager energy operator and Perceptual Wavelet Packets (PWP). The parameterized audio frames are fed into different probabilistic classifiers based on Gaussian Mixture Models (GMM) and Hidden Markov Models (HMM), following the OCC approach: GMM clustering, Universal GMM (UGMM) and universal HMM. Anomalous sounds are detected by evaluating the likelihood that they have been generated by the probabilistic majority class model, according to a pre-evaluated threshold. Although the events of interest are not artificially mixed with background noise, the hazardous situations are simulated with professional actors; thus, the gathered data cannot be strictly considered as collected in actual real-life conditions.

Following a similar approach, Aurino et al. apply OCC based on Support Vector Machines (SVM) to detect anomalous audio events within an automatic surveillance framework [43]. Specifically, the work was focused on the recognition of three types of burst-like acoustic anomalies: gun-shots, broken glasses or screams, which are defined and used to train one OCC-SVM per class a priori. The system follows a two-stage classification scheme by classifying the short audio segments (of 200 ms in length in the experiments) at the first level through an ensemble of OCCs and then aggregating their classification outputs into intervals of 1 s, which are reclassified using a majority voting strategy. The events of interest are artificially mixed with background noise acquired in indoor and outdoor environments. The audio events are parameterized using a set of typical audio features such as MFFCs, Fast Fourier Transform (FFT) or Zero Crossing Rate (ZCR), to name a few. The proposal yields similar performance compared to the multi-class alternative introduced in [54] focused on the same set of ANE. Finally, the authors highlight the flexibility and scalability of the classification architecture to include more anomalous events, leaving the inclusion of a preliminary detection stage between abnormal and background noise based on acoustic loudness for future work.

#### 2.2.2. Multi-Class Audio Event Detection

In [38], the authors present an HMM-based AED, which is evaluated on an event-annotated audio material from 10 different everyday environments: basketball, bus, office, restaurant, street, beach, car, hallway, shop and stadium with track and field events. The dataset is composed of 61 different categories, which are artificially mixed with ambient background noise at different SNRs (from −10–+10 dB in steps of 5 dB). The audio feature extraction is based on MFCC. The experiments, under a five-fold cross-validation scheme, show a recognition performance of 24% for real-life recordings at an SNR of 0 dB. Later, this group of researchers presented an unsupervised AED approach focused on the detection of overlapping audio events [55]. The proposal was evaluated on the same dataset, but using Non-negative Matrix Factorization (NMF) and parameterizing the audio input by FFT or Mel spectrum. A five-fold cross-validation scheme was also considered, but with the particularity that one fold was used as a development set for determining the most suitable number of clusters. The results outperform previous experiments and the supervised baseline in most of the everyday environments. In [56], a similar approach based on NMF and short-term FFT is considered as a preliminary study towards isolating the contribution of road traffic noise from acoustic measurements in urban sound mixtures. The results show an improvement in obtaining a reliable estimation of the traffic noise level without the influence of other noise sources. However, its implementation in a real-time monitoring system becomes very difficult since it is a computationally demanding technique. Furthermore, the conclusions are based on a very limited set of examples, which have been generated from synthetic mixtures.

In [57], Ntalampiras introduced an approach for acoustic surveillance in urban traffic environments following a multi-class AED approach. In this piece of research, the AED is based on a two-stage HMM-based classification system, which analyzes a multi-domain feature set, including again MFCC, LLD and PWP to consider time, frequency and wavelet domains. The work includes a database composed of nine audio classes: car, motorcycle, aircraft, crowd, thunder, wind, train, horn and crash. The samples are obtained from several professional sound effect collections assuring high quality without being affected by background noise. Moreover, the detection of crash incidents is also studied by merging those sound events with the rest of the classes at specific SNRs (i.e., 0, 5 and 15 dB). Thus, the experimental configuration of the proposal is far from that obtained in real-life recording conditions.

In [58], a real-life urban sound dataset named UrbanSound and its compact version UrbanSound8k are introduced. The dataset is composed of 10 low-level classes under the following taxonomy: construction (drilling and jack hammer), mechanical (air conditioner and engine idling), traffic (car horn), community (dog bark, children play and street music) and emergency (gun shot and siren), whose events are artificially mixed with the background noise considering various SNR values. The audio data are differentiated between foreground and background, depending on their acoustic (subjective) salience. In order to study the characteristics of both datasets, an AED is also implemented and tested following a 10-fold cross-validation scheme. The AED is based on MFCCs plus some derived statistics and some off-the-shelf classifiers provided by the Weka data mining platform. The experiments show that SVM and random forest present the best results. Moreover, the authors demonstrate the variability of the AED in terms of classification accuracy in relation to foreground and background noises. Recently, in [28], the authors have presented an AED following local and global features aggregated based on a mixture of experts model, which is also tested using the UrbanSound8k dataset.

In [26,32], the AED is designed to identify and locate a quite heterogeneous variety of sound events derived from hazardous situations, such as people screaming or gun shots, some of them being specifically related to traffic, such as horns, road accidents and types of vehicles. A three-node WASN is deployed in [32] to test the proposal, which takes advantage of ambisonic microphones and the AED on a centralized media server. The AED is based on quadratic discriminant analysis and neural networks as the classification approach, adjusting an N-dimensional density model to each sound class of interest. As for the acoustic parametrization, the authors discuss the consideration of low level signal features or psychoacoustic properties such as MFCC. Unfortunately, the authors do not provide any quantitative measure of the system performance in terms of acoustic event detection.

Foggia et al. have recently presented an adapted approach of their AED, focused on the detection of specific noise events, such as screams, glass breaking or gunshots [25] to detect anomalous sounds within urban traffic, such as tire skidding and car crashes on low-cost hardware platforms for urban surveillance [34]. In a nutshell, the proposal extracts acoustic low-level features of the input audio to capture the discriminant properties of the acoustic events of interest. A bag-of-words representation is used to perform AED after training a pool of SVM-based classifiers that consider different feature extraction techniques. The work provides a sensitivity analysis in order to evaluate the detection capability of the hazardous events at different distances from the microphone. The authors compare the results obtained with the 15 dB train and test scheme with hazardous events presenting −3 dB and −6 dB of the original audio signal. The results show a significant loss of system generalization capabilities, yielding the MFCC plus the proposed classification approach as the best performing configuration in terms of the ROC curve. Finally, it is worth mentioning that although the study is based on audio signals recorded in real-life environments, a synthetic audio database is generated by mixing these data with the two classes of hazardous road events of interest with a signal to noise ratio up to 15 dB.

Finally, in [33], a preliminary study on the detection of anomalous noise events within road traffic noise was developed and tested on a small synthetic database (several minutes of audio data). The database was built to simulate a real-life environment by gathering on-site recordings from the ring road surrounding the city of Barcelona (i.e., a suburban environment), artificially mixed with several ANE sounds obtained from Freesound project (http://freesound.org/) (at +6 dB or +12 dB with respect to the background RTN). The AED was implemented considering two classification approaches: *k*-Nearest Neighbor (*k*-NN) and Fisher Linear Discriminant (FLD) analysis; as well as two audio parametrization techniques: MFCC and Gammatone Cepstral Coefficients (GTCC) [59]. The best results were obtained for the GTCC-FLD configuration for ANEs with an SNR of +12 dB after a 10-fold cross-validation evaluation. Unfortunately, the conclusions drawn from this artificially generated dataset have proven to be non-generalizable to real-life conditions recently [40].

In most of the aforenamed papers, the authors claim to test their approaches in real-life environments. Nevertheless, typically an artificial picture of what can be found in actual real-life conditions is generated by means of building synthetic mixtures from real field or online repositories such as Freesound, or through the recreation of hazardous situations with professional actors, or even the modification of ANE distribution (e.g., the noise classes are truncated to a maximum of 1000 samples per class in UrbanSound and UrbanSound8k [28,58]). Moreover, the revised works are mostly evaluated in laboratory conditions or implemented in centralized servers, but not in the acoustic sensors themselves. Finally, when applied to traffic noise, they only focus on surveillance and not on reliable noise map generation in urban environments, which is the problem dealt with in this work.

## 3. Anomalous Noise Event Detector

In this section, we describe the proposed anomalous noise event detector designed to work in real-time on low-cost acoustic sensors of a WASN.

**Figure 1:**
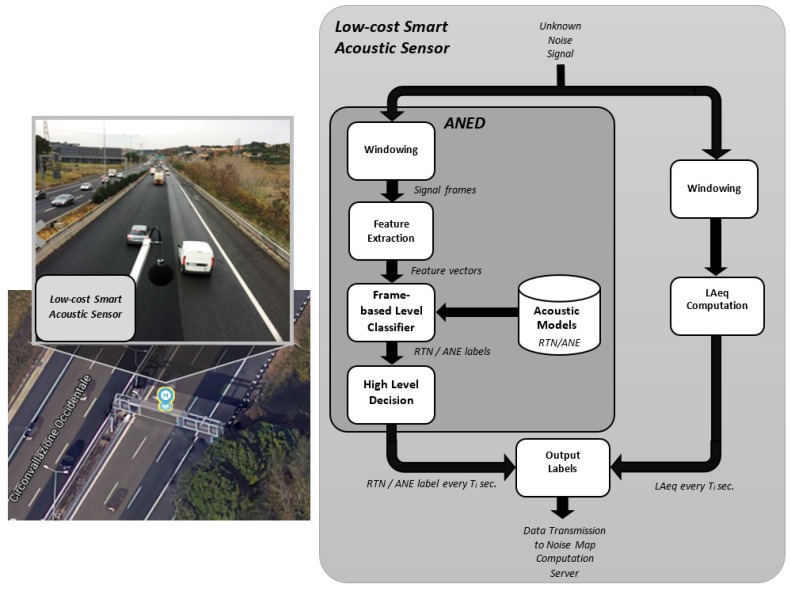
Example of the low-cost smart acoustic sensor positioning in a suburban environment (pictures loaned courtesy of ANAS S.p.A.) and the ANED functional block diagram.

Figure 1 shows an example of the placement of a low-cost smart acoustic sensor in a real-life operating environment, together with the block diagram of the main signal processing modules running on the acoustic sensor. It performs two basic functional operations: (i) the computation of the $LA_{eq}$ using a predefined integration period; and (ii) the computation of a binary label attributed to the type of input sound source (RTN or ANE) during the same period of time. These two values are updated in a central server in real time, in order to perform further computations to obtain $LA_{eq}$ values with the desired time intervals (e.g., 5 min, 15 min, 1 h, etc.), before generating the corresponding dynamic traffic noise maps. The ANED output label contains the typology of the sound source, which evaluates the $LA_{eq}$ values considering the binary labels (RTN or ANE) sent from each sensor of the WASN to entail the noise map of the monitored road infrastructures. This architecture minimizes the data transmission between the sensor and the server and minimizes the computational load of the central server due to the distributed evaluations in each sensor.

The ANED binary outputs are obtained following a two-level decision scheme. Firstly, the ANED classifies the input audio data between RTN and ANE at the frame-level based on a two-class detection-by-classification approach [20], after building the corresponding acoustic models from representative real-life data collected from both suburban and urban environments. Secondly, a high-level decision based on a majority vote criterion is obtained by fusing the frame-based decisions within the desired integration time $T_{i}$ to output a binary label (RTN/ANE) within that interval. In order to perform the frame-level decision, the ANED follows two main stages: feature extraction and classification. These two processes require a suitable real-time implementation to perform the signal processing algorithms on each of the low-cost acoustic sensor of the WASN. In Section 4, the ANED is adjusted and assessed considering the operating specifications of the DYNAMAP project on real-life urban and suburban acoustic data.

## 4. ANED Configuration and Assessment in Real-Life Urban and Suburban Environments

In this section, we adjust the ANED and assess its performance using the audio database designed in the framework of the DYNAMAP project, within its specifications and pilot areas (Section 4.1 and Section 4.2). Regarding the feature extraction and classification techniques, we consider the implementation of those approaches applied to similar problems found in the literature (Section 4.3) in order to select the optimal two-class novelty detection approach in terms of classification performance and computational costs (Section 4.4). Subsequently, the ANED performance is evaluated on continuous audio signals (Section 4.5), including the analysis of the high-level decision results.

### 4.1. DYNAMAP Project Operating Specifications

The DYNAMAP project envisages the deployment of a WASN in two pilot areas: a suburban area along the A90 ring road in Rome and an urban environment in the city of Milan. The real-time noise maps are computed using the measured acoustic data, and they will be depicted using A-weighted equivalent noise levels ($LA_{eq}$) on a Geographical Information System (GIS). In this project, the ANED detects and labels every $T_{i}=1$ second of data, informing the subsequent GIS [60] about the presence of ANE in the noise measurements. The frame length used is 30 ms, since this analysis window length has shown strong enough performance to detect anomalous noise events of an impulsive-like nature, as well as longer noise events [36]. Moreover, it has been agreed within the project specifications to consider as ANE the audio events that do not reflect regular road traffic noise in both urban and suburban environments, i.e., those that are not generated by vehicles’ engines or derived from the normal contact of the tires with the road surface [30].

Regarding the hardware platform, the DYNAMAP’s ARM-based acoustic sensor integrates a low-cost Class 2 microphone, properly tested to face simulated stress conditions [61], similar to situations with hard climate variations, and also an ARM-based mini PC that implements the real-time signal processing algorithms of the digitalized input audio signal [44].

### 4.2. Real-Life Urban and Suburban Acoustic Database

The real-life acoustic database comprises 9 h 8 min of labeled audio data, obtained by means of a recording campaign specifically conceived for the collection of anomalous noise events in the two pilot areas of the DYNAMAP project, covering both urban and suburban environments [30]. To that end, two sets of real-life recordings were performed using the DYNAMAP smart low-cost sensor and also a sound level meter (for reference purposes) at a 48-kHz sampling rate with 24 bits/sample: along the Rome A90 ring road (suburban scenario) and within the city of Milan (urban context). In the former scenario, up to five locations were acoustically measured at the A90 motorway portal structures, obtaining road noise profiles of fluid traffic during daytime. In the latter, up to 12 locations were sensed including from one-way roads with very-low traffic to roads with trams and railways and with fast traffic flow (multi-lane roads).

From these data, audio labels, including start and end points according to a predefined set of sound source categories—were generated by experts through listening to the full recordings in both areas. In the suburban area, 16,496 s of recorded audio were manually labeled as RTN, while 543 s were attributed to seven different ANE categories, such as sirens, noise of the portals’ structure derived from its vibration—typically caused by the passing-by of very large trucks, or horns or noise when trucks or vehicles with a heavy load passed over a bump. In contrast, 11,600 s were labeled as RTN, and 1932 s of audio were denoted as ANE in the Milan city urban context. In this case, also 2307 s were labeled as Background city noise (BCK), reserved to those recordings where it was difficult to identify the noise coming from road vehicles since they were composed of the background noise of the city. The ANE included a specific type of urban-like sounds, such as music, tramways and trains, sounds of doors or birdsongs, among others. During the recording campaign, some meteorological sounds were also observed, like wind and thunderstorms, but only in the urban scenario.

Finally, BCK and RTN observed in the urban case are considered to belong to the same class, which is denoted with the RTN label for simplicity. The main reason behind this decision is that the ANED is asked to deliver a binary output in order to compute the 1-s $LA_{eq}$ only within those periods that are not affected by the ANE. Furthermore, it is worth mentioning that ANE represents about 3.2% of the acoustic data in the suburban environment and 12.2% in the urban context, respectively. Among them, only 29% of ANE presents a significant acoustic salience of SNR ≥6 dB in the urban environment, a percentage that decreases dramatically to 1.85% in the suburban context. These values show the inherent complexity of the identification of ANE within real-life recordings due to its unbalanced nature and entail significantly lower acoustic salience with respect to synthetically-generated balanced databases the reader is referred to [30] for a detailed analysis of the considered acoustic database and its comparison with other artificial alternatives.

### 4.3. Feature Extraction and Classification

In this section, we briefly describe the audio features together with the classification techniques considered for the subsequent experiments designed to assess the ANED proposal.

Regarding the feature extraction, the Mel-Frequency Cepstral Coefficients (MFCC) [62] are considered to parameterize the input audio. The main reason for choosing these type of features is two-fold. On the one hand, it is a de facto standard used for the AED research community. On the other hand, MFCC can be efficiently computed in real time, which is an important aspect in a real-life computation context, such as the one analyzed in this work. Specifically, 13 MFCCs are obtained at a regular short time basis from signal frames of 30 ms in length.

As for the core machine learning technique employed to implement the ANED, we have taken into account representative approaches applied in similar AED problems: Discriminant Analysis (DA); Gaussian Mixture Models (GMM); Support Vector Machines (SVM); and also k-Nearest Neighbors (k-NN) as a non-parametric method for classification. All these machine learners have been previously optimized (see Appendix A) before their comparison using the aforementioned real-life urban and suburban acoustic database that is shown in Section 4.4.

Moreover, we also consider the OCC approach based on probabilistic novelty detection using a Universal GMM (UGMM) introduced in [36] for outdoor surveillance purposes as a baseline for the conducted experiments. In this case, only the acoustic model of the majority class, in our case the RTN, is computed using the available training data, and a simple thresholding process is applied during the testing phase to classify input frames as RTN or ANE. A test feature vector is considered as novel when the probability obtained after its evaluation using the previously trained RTN-based UGMM falls below a predefined threshold, computed as the minimum probability of the training audio samples.

### 4.4. Selection of the ANED Core Two-Class Classifier at the Frame-Level

In this section, the performance of the aforementioned classifiers is evaluated at the 30-ms frame-level considering MFCC extracted with 50% of window overlap for each of the two analyzed scenarios (urban and suburban).

The performance of the studied classifiers is compared to the ground truth (elaborated with manual labels) in terms of the F1 macroaveraged measure (also named as the F-score with class-based averaging in [29]) of the two audio categories (RTN and ANE). The F1 measure of a given class is defined as the harmonic mean of the recall (R) and precision (P) (see Equation (1)). Recall is the number of True Positives (TP) divided by the number of real examples of a given class or the sum of true positives plus the False Negatives (FN) (see Equation (2)). Precision is the number of TP divided by the number of examples classified in that class or the sum of true positives plus the False Positives (FP) (see Equation (3)). Finally, the F1 macroaveraged measure is defined as the mean between the F1 measures of both the RTN and ANE classes (see Equation (4)).

(1) $$F1=\frac{2RP}{R+P}$$

(2) $$R=\frac{TP}{TP+FN}$$

(3) $$P=\frac{TP}{TP+FP}$$

(4) $${F1}_{Macro-av}=\frac{{F1}_{ANE}+{F1}_{RTN}}{2}$$

For each scenario and classification technique, a four-fold cross-validation scheme has been considered to obtain statistically meaningful results from a machine learning point of view. In order to ensure proper diversity of the generated cross-validation partitions, a random permutation of the feature vectors of each category (RTN and ANE) has been generated before the fold-based partition.

The chosen classifiers configuration is based on the previous study presented in Appendix A, being the following: DA with quadratic discriminant function; KNN with K=1; SVM with the radial basis function kernel; and GMM with 256 components for both acoustic models (RTN and ANE). The baseline UGMM classifier is set also with 256 Gaussian components for a fair comparison with the GMM counterpart.

#### 4.4.1. Analysis of the Results

**Figure 2:**
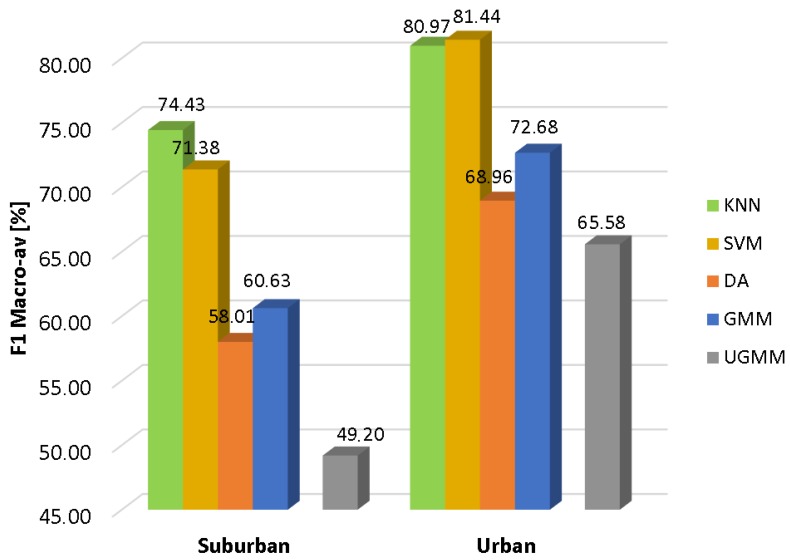
Classification performance of the studied classifiers for the suburban and urban scenarios.

In Figure 2, the results of the conducted experiments for the suburban and urban scenarios are depicted respectively in terms of the macroaveraged F1 measure. It can be observed that all machine learning algorithms follow a similar performance pattern in both scenarios, with the urban case attaining higher classification scores. Furthermore, there is a similar behavior regarding the classifier performance for a given scenario, KNN and SVM being the two classifiers with the highest reliability (KNN being the optimal classifier for the suburban environment, while SVM is the best option for the urban one), followed by GMM, DA and UGMM, in decreasing order. It is noticeable that UGMM obtains poor quality decisions in the suburban environment.

Moreover, all optimized classifiers outperform the UGMM baseline in both environments within the our-fold cross-validation scheme. Specifically, GMM outperforms the F1-score of the UGMM by 23.2% and 10.8% in the suburban and urban pilot scenarios, respectively, while the KNN improves the UGMM F1 values by 51.3% and 23.5% in both environments, respectively, and finally, the SVM attains a relative improvements of 45.1% and 24.2% in comparison with UGMM in the same scenarios.

Furthermore, specific conclusions can be derived when comparing the two considered environments. In general terms, the classification results are better for the urban database than the suburban one (around an average of 19% higher F1-score across classifiers). This can be explained by the fact that the ANEs recorded in the urban database present larger SNR values than the ones collected in the suburban environment on average [30], providing also the nature of the ANE data’s higher separability from the background road traffic. For instance, ANEs with high acoustic salience recorded in quiet background noise (e.g., car horns in a one-way street) can be more easily detected than when the same anomalous audio event comes from a highway with fluid traffic conditions in the suburban environment. As previously mentioned, the urban database comprises about 29% of ANEs with high salience (e.g., SNR ≥ 6 dB), while this number decreases down to 1.85% in the suburban case. Nevertheless, further research should be conducted to evaluate in detail these preliminary conclusions.

From the analysis of these results, it can be concluded that the discrimination of anomalous noise events using the considered ANED binary-based classification scheme outperforms the baseline UGMM one-classification scheme, reaching up to 74.4% and 81.4% macroaveraged F1 values for the suburban and urban scenarios, respectively, for the best classifier in each scenario under the four-fold cross-validation scheme. Moreover, the results also demonstrate the performance differences when both environments are compared.

#### 4.4.2. Computational Cost Analysis

The last stage of the validation is the real-time sensor implementation computational load evaluation. The algorithm that best satisfies the trade-off between computational cost (allowing for real-time operation) and good F1-scores has to be implemented in a low-cost smart acoustic sensor developed by Bluewave [44,61]. This sensor is based on an ARM architecture [63], and it is sized to compute the basic acoustic signal processing algorithms in order to detect ANEs in the sensor, assuming that the algorithms require a moderate computational load. The sensor also evaluates the $LA_{eq}$ every 1 s and sends both the $LA_{eq}$ value and its label (RTN/ANE) to the central server (see Figure 1). For the sake of comparison, the ANED initial configuration used in the Linux-based SDK programming of the sensor computes 13 MFCC every 30 ms, classifying each input audio frame using a GMM trained with 32 Gaussians per class, generating the final output after the high-level binary decision every 1 s. The computational results show the feasibility of the real-time computation of the labels over the low-cost designed sensor, as running the ANED only increases the microprocessor load by about 14%. The same configuration has been tested on a desktop PC with advanced features (Intel Core i7-4710 CPU 2.5 GHz, with 32 GB RAM), and the computational load of the algorithm is nearly negligible with respect to the entire performance of the computer, with the mean CPU load calculated to be 0.01614%.

In the following paragraphs, the classifiers previously compared in terms of classification performance are tested in terms of their computational complexity to evaluate the feasibility of their implementation in the smart low-cost acoustic sensors of the DYNAMAP project. The computational load is tested in the aforementioned desktop PC for each of the two machine learning phases (train and test), as a preliminary evaluation over a high computation device. To that effect, we compute the required CPU load by dividing the mean training and test time for each fold of the four-fold cross-validation analysis scheme by the corresponding duration of each training or testing dataset (considered as the real-time RT(%) estimator). Classifiers have been implemented as a prototype using MATLAB toolboxes.

**Figure 3:**
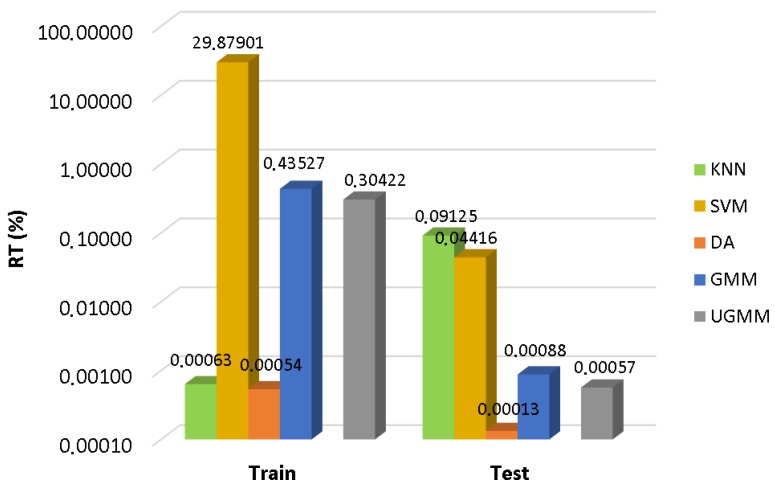
Computational cost comparison among the considered machine learning approaches.

As can be observed in Figure 3, SVM entails the highest computational cost in the training phase, followed by the GMM and UGMM classifiers. However, training time is not a relevant factor in the selection of the optimal classifier to operate in the WASN sensors. In the test phase, the most efficient classifier is the DA followed by UGMM and GMM. The KNN classifier obtains the worst testing performance in terms of computational load (as it requires comparing the complete training dataset with each of the input feature vectors of the test dataset), followed by SVM. Although KNN and SVM yield the best scores in terms of F1 measures in suburban and urban scenarios, respectively, these results make us discard them for the real-time classifier running in the acoustic sensors of our WASN. The main reason is that the estimation of the computational load of both classifiers in the ARM-based sensor, from the performance observed for both the sensor and the desktop PC when ANED is configured with a GMM-32 classifier, does not attain real-time operation. As a result, the two-class GMM based classifier is selected for the subsequent analyses, as it yields a good trade-off between performance, in terms of macroaveraged F1-scores, and computational cost, measured as the mean test computational load, both under the four-fold cross-validation scheme.

### 4.5. Analysis of the ANED Output after the High-Level Decisions

In this section, an assessment of the ANED at the high-level $T_{i}=1$ second decision is performed, using the same configuration tested in the sensor. The performance of the ANED after the high-level decision is evaluated considering both the two-class GMM-based classifier, as the selected core classifier after the 30 -s frame-level experiments, and the one-class UGMM, as the baseline classification technique with the same number of Gaussian components. In order to allow the comparison between both classifiers, the ANED high-level decision is obtained by means of a majority vote of all the fame-level outputs within the 1-s interval of integration. Then, a 1-s interval is labeled as ANE if 17 or more signal frames within this period of 33 frames are labeled by the frame-level classifier as ANE, while the output label is RTN otherwise. Apart from permitting the integration of the ANED system to the time requirements of a WASN, i.e., producing output labels at the same time intervals of the equivalent noise level computation, this smoothing process could limit the detection of impulsive-like (e.g., a door sound) or intermittent audio events, but conversely, it can improve the detection probability of continuous and longer sound events (e.g., sirens or airplanes), generally, having a higher impact on the equivalent noise level computation due to its duration and SNR. Moreover, in the following paragraph, we compare the reliability of the frame-based decisions with the high-level decisions in the two analyzed scenarios.

F1 measures for frame-based level decisions are obtained using the ground truth labels from the manual labeling process of the audio database. However, for the computation of F1 measures of high-level decisions, the ground truth labels at the frame-based level are used to compute the high-level ground truth labels by using the same majority voting scheme. Then, when a 1-s period of time is labeled as ANE for the ground truth, it means that it contains more frames of ANE than RTN.

In Section 4.4, the different frame-based classification approaches have been assessed using a four-fold cross-validation scheme ensuring data homogeneity in each fold by means of data randomization. However, this scheme does not allow the evaluation of the ANED performance in real operation conditions after the high-level decision stage. To do so, we evaluate the high-level decision of the ANED in a continuous audio signal every second, following a Leave-One-Out cross-validation scheme (LOO). The audio coming from one specific recording site has been used for testing, while the remaining 11 recordings are used to train the corresponding classifier (GMM or UGMM) for the same suburban or urban environment (in both environments, a total of 12 recordings were obtained). As a consequence, this evaluation scheme is more demanding in terms of AED and classification compared to the four-fold cross-validation scheme, were the test data have been randomly selected across the RTN and ANE classes (e.g., some partitions contain certain types of anomalous noise events that others scarcely contain).

**Figure 4:**
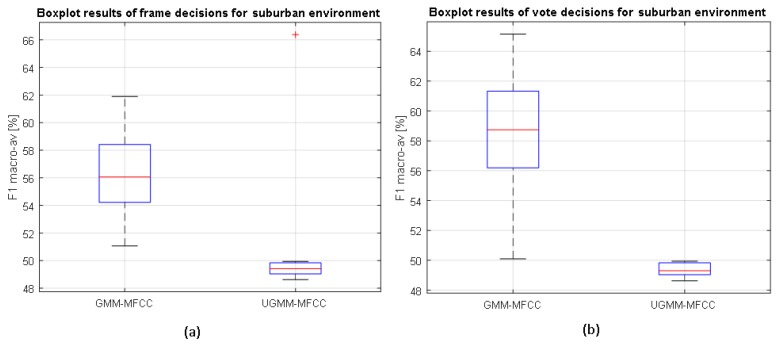
Boxplots of the classifiers’ performance at the (**a**) frame level and (**b**) high level on the suburban environment considering the binary-based GMM and UGMM classifiers and MFCC features, as well as following a leave-one-out cross-validation scheme.

**Figure 5:**
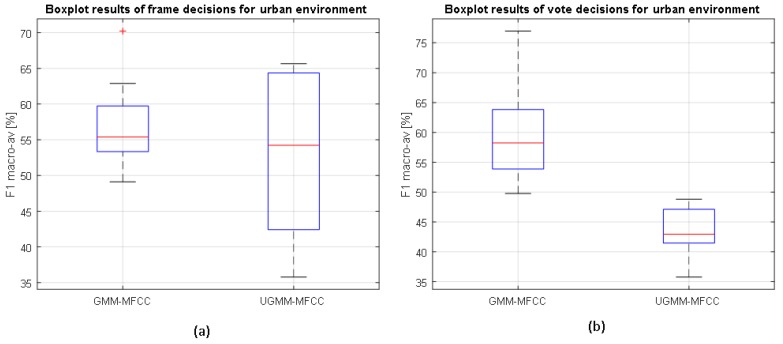
Boxplots of the classifiers’ performance at the (**a**) frame level and (**b**) high level on the urban environment considering the binary-based GMM and UGMM classifiers and MFCC features, as well as following a leave-one-out cross-validation scheme.

The results of the LOO scheme are depicted in Figure 4 and Figure 5, showing the classification distributions (using boxplots) of the binary-GMM and UGMM classification schemes in terms of the macroaveraged F1 measure accounting for 12 iterations. Binary-GMM clearly outperforms UGMM in all the conducted tests at both test sites, despite the F1-macroaveraging value worsening the results obtained in the four-fold scheme (see Section 4.4). The reduction of classification performance has been reported in previous studies (e.g., see [59]), mainly due to the variate particularities of each of the recording sites used to test the system. Moreover, the differences between both distributions are evaluated statistically. For each pair of compared distributions, a Shapiro–Wilk parametric hypothesis test of composite normality has been run, and all analyzed configurations have passed the test with a standard significance level of p≤0.05. Then, a paired-sample *t*-test has been computed to evaluate if the differences between the different configurations are significant, also considering p≤0.05.

#### 4.5.1. Results in Both Suburban and Urban Environments

As can be observed from Figure 4, the binary-GMM classifier clearly outperforms UGMM, a difference that is statistically significant for both types of decisions (frame and high level). Moreover, the classification performance improves with respect to frame-based decisions after the high-level decision in the case of the binary-GMM, which is also statistically significant according to the *t*-test. In particular, the binary-GMM relatively improves the baseline UGMM macroaveraged F1-measure by 18.7% and 31.8% for suburban and urban environments, respectively. Therefore, it can be concluded that the ANED using the binary-GMM considering two probabilistic models (one for RTN and another for ANE) significantly outperforms the UGMM baseline at both the frame and high-level decisions in the suburban environment.

In the urban environment, the binary-GMM classifier yields median macroaveraged F1 measures higher than the UGMM counterpart at the frame level (see Figure 5). However, those differences are not statistically significant. On the contrary, it attains statistically significantly better results than UGMM for the high-level decisions.

From these results, we can conclude that the binary-GMM approach could be considered as a good core classifier of the ANED for both suburban and urban environments.

#### 4.5.2. Comparing High-Level vs. Frame-Level ANED Decisions

**Figure 6:**
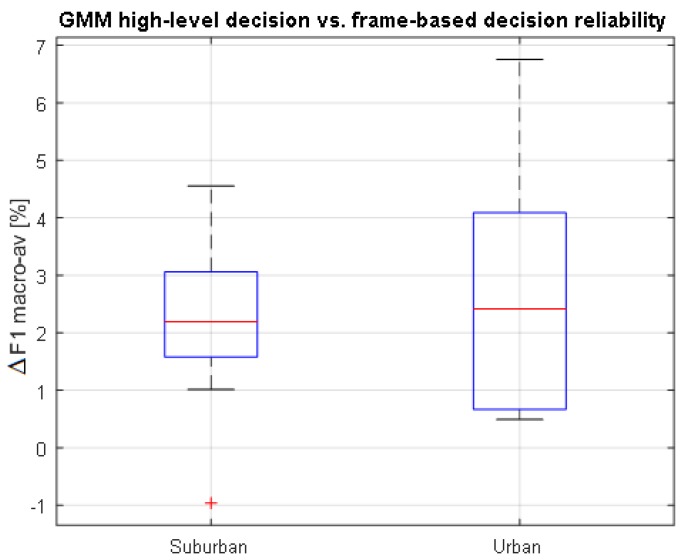
Boxplots of differences of the classifiers’ performance between high-level and frame-based decisions using the binary-based GMM classifier following a leave-one-out cross-validation scheme. Results are shown for suburban (left-side) and urban environments (right-side), separately.

The integration of ANED within a WASN devoted to road traffic noise mapping demands a binary label for each integration time interval $T_{i}$ where the A-weighted equivalent noise level ($LA_{eq}$) has to be measured; in this work, $T_{i}=1$ s. To have a clearer picture of the performance improvement that the high-level integration obtains with respect to the frame-level decisions, a specific comparison has been conducted based on the binary-GMM ANED core classifier. To that end, the macroaveraged F1 measures obtained by all the frame-level decisions that are included within a 1-s period are subtracted from the high-level decision that accounts for the same period of time, conducting a subsequent statistical analysis for the LOO evaluation scheme. As can be seen from Figure 6, the high-level decisions’ accuracy is higher than that of the frame-based outputs in the urban environment. On the other hand, however, the increment of high- vs. frame-level decisions is lower for the suburban scenario, although most of them are positive. Therefore, we can conclude that the high-level decisions act as a positive smoothing post-processing stage, yielding 3.8% and 4.4% of relative mean improvement of the F1 measure for the suburban and urban environments, respectively.

## 5. Discussion

In this section, we discuss several open questions derived from the ANED proposal developed in this work. Key issues related to the feature extraction and the core classifier are discussed, taking into account the results obtained in this work. We also detail some considerations about the characteristics of the real-life dataset used to perform the experiments and its impact on the ANED evaluation.

### 5.1. Feature Extraction Considerations

After the real-life dataset analysis [30], this work focused on the design of the ANED core audio event detection and the classification algorithm both in terms of performance and computational load to ensure its real-time implementation in the smart low-cost acoustic sensors of a WASN devoted to road traffic noise mapping. To that effect, we have parameterized the audio data using MFCC [62], a de facto standard in terms of feature extraction for speech and audio recognition.

Two key issues should be considered in the development of the ANED when considering feature extraction: the first one a more detailed study of the AED performance variation depending on the nature of the sounds to be identified as observed in the urban and suburban scenarios; this requires a specific comparison of feature extraction techniques [29,64]. The second issue is to keep working on the inclusion of perceptually-based parameters such as MFCC, which may improve the performance of the ANED if they respond to human hearing in a more precise way.

The first goal, mainly focused on the feature extraction, should lead us to consider the signal time evolution, which in this paper is solved by the majority voting of all the 30-ms frame-level decisions contained in a 1-s ANED label evaluation. Spectro-temporal features, widely studied in the birdsong recognition environment [65] due to the nature of the repetitiveness of the acoustic signal to be identified, can be also considered, because both classes (ANE and RTN) are composed of very heterogeneous types of acoustic signals. The consideration of both the spectrum and the time variation of the signal could take into account both impulse-like signals and long-lasting sounds and extract information of time pseudo-periodic signals.

The second goal aims to integrate other techniques of perceptual parameterization in the feature extraction stage [64]. For instance, we can compare MFCC and GTCC [59], which are also based on biologically-inspired filter adjusting and have shown good results in similar applications. Moreover, we can also consider perceptual wavelet packets [66], which have shown better results in other environments when compared with MFCC (and even with wavelets and MPEG-7 audio-based representations).

The combination of the temporal information on the acoustic signal together with the change of paradigm in terms of perceptual descriptors is expected to improve the obtained MFCC baseline performance (evaluated in terms of the macroaveraged F1 measure) of the ANED algorithm. However, a minimum increase of the computational load of the entire parametrization process must be guaranteed to maintain the ANED computation in real-time within the ARM-based acoustic sensor.

### 5.2. Classification Considerations

The binary-based Gaussian mixture model has yielded a good overall performance in the tests conducted in this paper, improving the universal GMM one-class classifier considered as a baseline in both testing configurations, i.e., the four cross-fold and leave-one-out cross-validation schemes. GMM has obtained better results than UGMM as a good trade-off between classification performance and computational costs with real-life data.

Despite the good performance of the GMM in this first ANED approach, there is room for improvement in the robustness of the 30-ms classification. For that purpose, the authors consider that the potential next steps should be both the optimization of the preliminary proposal and the comparison of the GMM performance with other machine learning approaches. To this aim, although Deep Neural Networks (DNN) have yielded good performance in multi-class AED-related applications [31], they need a huge amount of labeled data to perform the training satisfactorily. Actually, this is a strong requirement of DNN, which, in the problem at hand may become an unfeasible challenge if a large enough ANE data sample has to be collected in real-life scenarios due to its unbounded nature. Notice that enlarging the ANE class with synthetic mixtures has been demonstrated to be suboptimal [30].

After the 30-ms frame-level decision, the system discriminates between RTN and ANE by means of a higher-level decision along a 1-s temporal window from all the 30-ms frame-based binary decisions made by the short-term classifier (see the ANED block diagram in Figure 1). This stage has been implemented by means of a simple, yet effective majority voting scheme [43], which has yielded for the ANED a more stable output (as discussed in Section 4.5.2). The reason for such improvement with regard to the frame-level decisions can be due to the major presence of continuous and lengthy ANEs (see Figure 9 in [30]).

### 5.3. Dataset Characteristics and Their Influence on the ANED Evaluation

In this work, the design of the ANED system has considered raw real-life audio data. On the one hand, the results have shown that including representative ANE for training the two-class based ANED has enabled us to obtain better results than ignoring them through the one-class novelty detection alternative. On the other hand, the high diversity of ANE duration and SNR values collected entail a very challenging operating scenario as observed in the performances obtained within the LOO evaluation scheme. It is worth mentioning that the experiments in this work have been carried out far from the prototypical conditions considered in similar previous investigations (e.g., training and assessing their approaches using a limited and regularly spaced set of SNR values). For instance, it seems reasonable to argue that the lower performance of the tested classifiers in the suburban scenario with regard to the urban one can be attributed to the lower values of ANE signal-to-noise ratios, in which only 1.85% of the collected events show an SNR ≥ 6 dB.

Also as a consequence of using raw real-life data, the dataset presents a high class imbalance between the two main categories [30], RTN and ANE, which the ANED is asked to discriminate. For the correct evaluation of the test results, we have considered the F1 macro-averaging measure, since it ensures non-biased comparisons between differently populated categories (which would happen when evaluating a simple recognition accuracy). Nevertheless, the classifiers training performed using the complete dataset can still bias their performance due to this imbalance. To that effect, several strategies can be adopted to obtain more balanced datasets, but they should be chosen accurately to ensure that they reflect real-life complexity and avoid artificially distributed data.

The ANED using a binary-based GMM classifier has shown substantially better results in the four-fold cross-validation experiments than in the LOO test configuration. Similar significant reductions have been reported by our research group in previous studies (e.g., see [59]), mainly due to the variate particularities of each of the recording sites used to test the system. In a preliminary analysis, we have observed that the ANE class data heterogeneity across the recording sites may cause some folds to present pretty good performance while others yield worse results. However, a detailed study on the occurrences of the different ANE and their nature (e.g., long, short, salient, etc.) for each recording site could lead us to firmer conclusions about the dependency of the LOO results of the nature of the site under test.

One of the main novelties of this study relies on training and testing the ANED with labeled audio data from real-life urban and suburban environments, where the ANE are deeply mixed with background noise and road traffic noise; so, no artificial mixing process, which could distort the final result of the samples of the dataset, is needed. Artificial mixing permits a high degree of control of what is being simulated and an exact monitoring of the time limitation of each of the noise events to evaluate. However, configuring a synthetic mix that truly reflects a real-life audio scene requires knowledge obtained through in-depth analysis of audio databases that reflect the true diversity of the nature of the problem. Nevertheless, the specific nature of ANE makes it difficult to collect the large diversity and heterogeneity of the anomalous noise events that can be found in urban and suburban environments. For instance, only a few sites in the urban context of the used database contain night recordings, nor do the include diverse meteorological conditions (e.g., wet pavement, hail, snow, strong wind, etc.), although the suburban environment is less diverse than the urban environment in terms of ANE. More work needs to be done in the way audio databases are used for training and testing the two-class ANED, which in this work have been used as they are found in real-life environments.

## 6. Conclusions and Future Work

In this work, we have introduced an anomalous noise event detector based on a two-class audio event and classification approach to be implemented on low-cost acoustic sensors of a WASN devoted to road traffic noise mapping. The conducted experiments, considering the DYNAMP’s project operating specifications, have shown the feasibility of the proposal both in terms of computational cost and classification performance using raw real-life data collected from both suburban and urban environments. Moreover, the results prove the viability of the two-class classification scheme to detect ANE with respect to the one-class baseline within a database that includes a representative set of road traffic noise and anomalous noise events in their actual distributions in a real environment.

In particular, the GMM has been selected as the best core binary classifier for the ANED at the 30-ms frame-level decision, according to the results obtained following a prototypical four-fold cross-validation scheme. The experiments have shown that the binary-based GMM classifier relatively improves the macroaveraged F1 measure of the alternative UGMM-based one-class classifier for the 1-s high-level decision by 18.7% and 31.8% for suburban and urban environments, respectively. These results have been obtained after running the ANED on continuous audio streams under a leave-one-out cross-validation scheme. Moreover, it is worth mentioning that the high-level decisions improve the frame-level decisions, yielding 3.8% and 4.4% of relative mean improvements in terms of F1 values for the suburban and urban environments, respectively; thus, validating the viability of the majority voting scheme as a smoothing post-processing stage. Finally, the experiments have also shown that the type of environment and evaluation scheme clearly influences the ANED performance, with the suburban environment being the most challenging because of the type of collected ANE and traffic conditions (e.g., yielding a 74.4% macroaveraged F1 score in front of the 81.4% for the urban environment with the best performing core classifier under the four-fold cross-validation scheme). Nevertheless, we leave the detailed analysis of the data distribution and the homogeneity of the audio events collected in both environments, together with the ANED optimization in terms of feature extraction as discussed previously for future works.

Furthermore, since the WASN deployment of the DYNAMAP project also entails the implementation of the ANED for low capacity μC-based sensors, it will be necessary to adapt the ANED designed for the ARM-based nodes to operate in real time in those low-capacity sensors powered by a solar panel. In this context, the ANED should be redesigned to reduce its computational cost as it will be critical to the proper functioning of the system, since the μC-based sensor should be able to calculate, not only the ANED binary label, but also the $LA_{eq}$ every second and, after that, send both values to the central system in real time. We plan to work towards this goal as the next step in our research. Finally, another aspect that is left for future work is the study of the influence of the sensing conditions, specifically in regard to the positioning of the sensors’ microphone in relation to the sound sources. This question should be also addressed in the aim of studying the sensitivity of the ANED to installation procedures and whether specific adaptation techniques should be considered to minimize the need for new data collection and labeling to reduce the cost of the DYNAMAP’s WASN deployment.

## Figures and Tables

**Figure A1:**
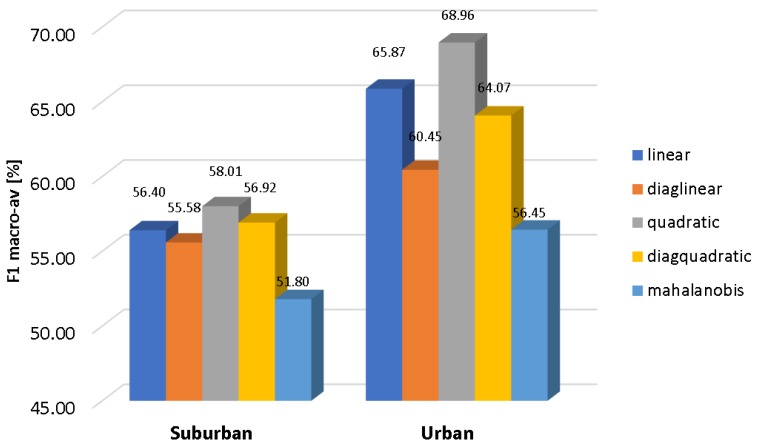
DA optimization results using the real-life suburban and urban databases, respectively. A different discriminant function has been used for each experiment (see the figure legend).

**Figure A2:**
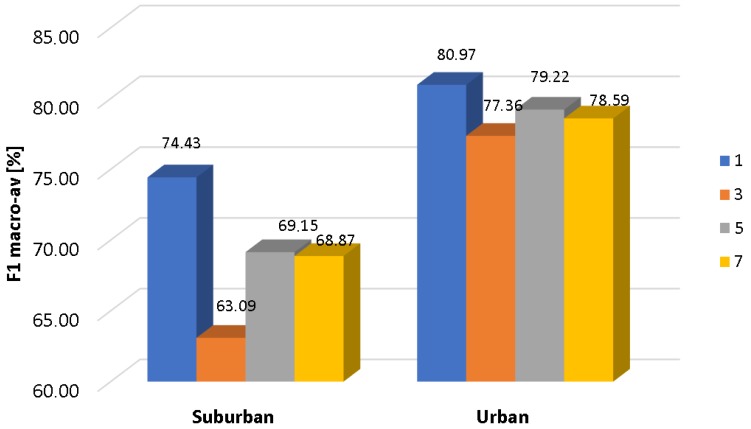
KNN optimization results using the real-life suburban and urban databases, respectively. A different value of the number of neighbors *K* has been used for each experiment (see the figure legend).

**Figure A3:**
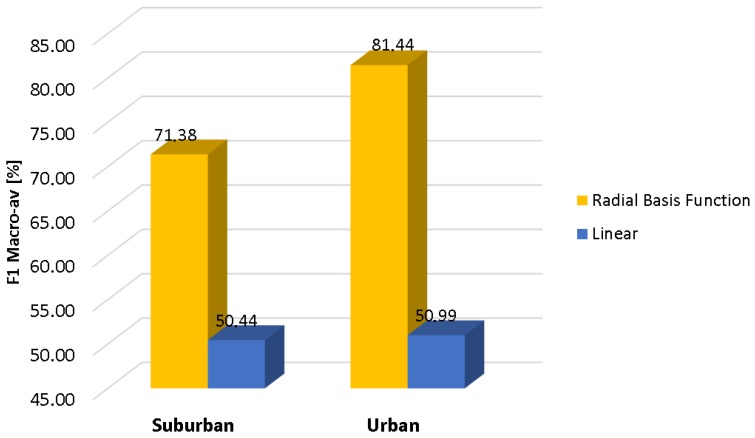
SVM optimization results using the real-life suburban and urban databases, respectively. A different type of kernel has been used for each experiment (see the figure legend). This result demonstrates that the addressed classification problem is clearly non-linear.

**Figure A4:**
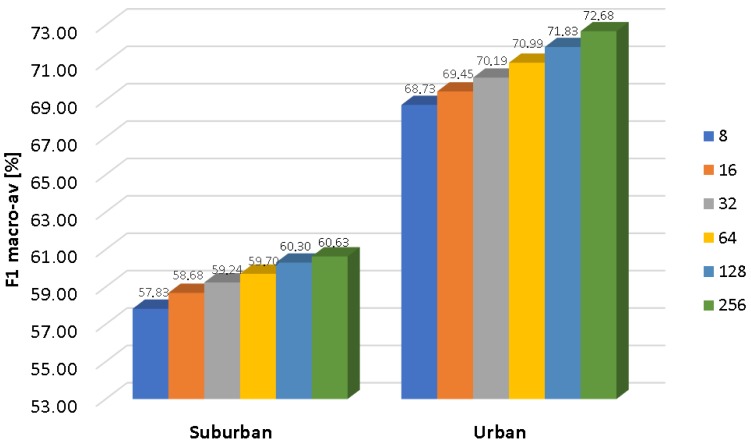
GMM optimization results using the real-life suburban and urban databases, respectively. In each experiment, a different number of Gaussian components has been set, which is shown in the legend.

## Abbreviations

The following abbreviations are used in this manuscript:

μC	MicroController
AED	Acoustic Event Detection
ANE	Anomalous Noise Event
ANED	Anomalous Noise Event Detector
ARM	Advanced RISC Machine
BCK	Background noise
DA	Discriminant Analysis
DNN	Deep Neural Network
FFT	Fast Fourier Transform
FN	False Negatives
HMM	Hidden Markov Models
GIS	Geographical Information System
GMM	Gaussian Mixture Models
GTCC	Gammatone Cepstral Coefficients
k-NN	k Nearest Neighbor
LLD	Low Level Descriptors
LOO	Leave-One-Out cross-validation scheme
MFCC	Mel Frequency Cepstral Coefficients
NMF	Non-negative Matrix Factorization
NN	Neural Network

OCC	One-Class Classifier
PWP	Perceptual Wavelet Packets
ROC	Receiver Operating Characteristic
RTN	Road Traffic Noise
SNR	Signal-to-Noise Ratio
SVD	Support Vector Machine
TP	True positives
UGMM	Universal Gaussian Mixture Models
WASN	Wireless Acoustic Sensor Network

## Appendix A. Classifiers Optimization

This section presents the optimization of the different machine learning algorithms (DA, KNN, GMM and SVM) considered as candidates for the classifier module of the ANED. The following parameters have been optimized for each of them considering the studies found in the related literature, namely: (i) the discriminant function for the DA as *linear*, which fits a multivariate normal density to each group, with a pooled estimate of covariance (this is, in fact, the Fischer linear discriminant classifier), *diaglinear*, similar to the linear function, but with a diagonal covariance matrix estimation, known as the naive Bayes classifier, *quadratic*, which fits multivariate normal densities with covariance estimates stratified by group, *diagquadratica* similar approach to quadratic, but with a diagonal covariance matrix estimation, and *mahalanobis* that uses Mahalanobis distances with stratified covariance estimates; (ii) the number of *K* neighbors of the KNN classifier, considering the following sweep of values: 1, 3, 5 and 7, and Euclidean distances; (iii) the type of kernel used for the SVM classifier being either *linear* or based on the radial basis function; and finally, (iv) the number of Gaussian components used in both acoustic models (RTN and ANE) of the GMM, evaluating an exponential sweep of 8, 16, 32, 64, 128 and 256 components.

The optimization of classifiers has been addressed considering the same methodology described in Section 4.4, following a four-fold cross-validation strategy assuring a random distribution of both RTN and ANE feature vectors in each fold or partition and obtaining the F1 macroaveraged measure at the frame level. The experiments have been repeated for each classifier configuration and also for each type of context (suburban and urban) using the corresponding audio database.

In Figure A1, the result of the discriminant analysis optimization is depicted. As can be observed, the *quadratic* discriminant function is the one that attains the highest F1 macroaveraged value for both environments, followed by the *linear* (Fischer linear discriminant) configuration.

Figure A2 shows the result of the KNN optimization process. The best results are obtained in both types of environments when using K=1, showing significantly better results with respect to the rest of the tested configurations in the suburban environment than in the urban one.

As can be observed from Figure A3, the radial basis function kernel obtains pretty good classification results for the SVM classifier (in fact, these are the best results in terms of the F1-measure for both scenarios and for all types of classifiers explored in this work), while its *Linear* counterpart presents dramatically lower performances.

Finally, the results of the GMM optimization process are depicted in Figure A4. We can observe a slightly constant improvement every time the number of components is doubled, obtaining the best performance when considering 256 Gaussian components per class, which also fulfills the computational resources of the considered low-cost smart acoustic sensor (see Section 4.4). It is of note that real-time operation is not achieved when using 512 Gaussians per class.
